# Reply

**DOI:** 10.1590/0004-282X-ANP-2021-0132r

**Published:** 2021-05-08

**Authors:** Gustavo Leite FRANKLIN, Brunna Nicole Goulart Vitória PEREIRA, Nayra de Souza Carvalho LIMA, Francisco Manoel Branco GERMINIANI, Carlos Henrique Ferreira CAMARGO, Paulo CARAMELLI, Hélio Afonso Ghizoni TEIVE

**Affiliations:** 1 Universidade Federal do Paraná, Hospital de Clínicas, Departamento de Medicina Interna, Serviço de Neurologia, Curitiba PR, Brazil. Universidade Federal do Paraná Universidade Federal do Paraná Hospital de Clínicas Departamento de Medicina Interna Curitiba PR Brazil; 2 Hospital da Cruz Vermelha, Departamento de Neurologia, Curitiba PR, Brazil. Hospital da Cruz Vermelha Departamento de Neurologia Curitiba PR Brazil; 3 Universidade de Vila Velha, Vila Velha ES, Brazil. Universidade de Vila Velha Universidade de Vila Velha Vila Velha ES Brazil; 4 Universidade Federal do Paraná, Hospital de Clínicas, Programa de Pós-Graduação em Medicina Interna, Grupo de Doenças Neurológicas, Curitiba PR, Brazil. Universidade Federal do Paraná Universidade Federal do Paraná Hospital de Clínicas Programa de Pós-Graduação em Medicina Interna Curitiba PR Brazil; 5 Universidade de Minas Gerais, Faculdade de Medicina, Departamento de Clínica Médica, Grupo de Pesquisa em Neurologia Cognitiva e do Comportamento, Belo Horizonte MG, Brazil. Universidade Federal de Minas Gerais Universidade de Minas Gerais Faculdade de Medicina Departamento de Clínica Médica Belo Horizonte MG Brazil

Dear Editor,

The contribution to the published article "Neurology, psychiatry and the chess game"^1^ was extremely rich, and it was very interesting to learn more about the life of the Mexican chess player Carlos Torre^2^. Due to space limitations, we were unable to include many personalities, such as the one mentioned, in our manuscript. Also, in Brazil, one of the greatest chess players of all time, Henrique Costa Mecking, better known as "Mequinho", was a leading exponent of the game, having reached third place worldwide, behind Anatoly Karpov and Viktor Korchnoi, in 1977. He was diagnosed with a neurological disease - myasthenia gravis - and had to withdraw from tournaments in 1978.

We are delighted and thankful for the valuable contribution of the letter by Jimenez-Ruiz and colleagues. It was a pleasure to know about the fascinating history of Carlos Torre.
